# All the Routes for Laparoscopic Liver Segment VIII Resection: A Comprehensive Review of Surgical Techniques

**DOI:** 10.3389/fonc.2022.864867

**Published:** 2022-04-01

**Authors:** Alessandro Anselmo, Bruno Sensi, Giulia Bacchiocchi, Leandro Siragusa, Giuseppe Tisone

**Affiliations:** Hepato-Pancreato-Biliary (HPB) and Transplant Unit, Department of Surgical Science, University of Rome Tor Vergata, Rome, Italy

**Keywords:** segment VIII, hepatobiliary surgery, laparoscopic liver resection (LLR), liver anatomy, hepatocarcinoma (HCC), liver cancer (LC)

## Abstract

Liver surgery is highly demanding for anatomical, physiological and technical reasons, and minimally invasive approaches have been implemented at a slower rate. Today, laparoscopic liver resection is a standard of care in many occasions, yet specific operations remain particularly challenging and generally performed in open surgery. In particular, SVIII resection may be considered one of the most difficult due to anatomical characteristics including its sub-diaphragmatic position, the deep-lying Glissonean pedicle and the close contact with the inferior vena cava and right and middle hepatic veins. Many techniques have risen to overcome technical difficulties, and today laparoscopic SVIII resection has been demonstrated to be feasible. This review provides a complete picture of current approaches, focusing on all techniques reported so far with critical appraisal of each, discussing and explaining benefits and pitfalls.

## 1 Introduction

Liver surgery is highly complex due to anatomical and physiological reasons, and therefore progress has historically lagged behind other abdominal surgeries. Nonetheless, pioneers in the 50s have made liver resection possible, and the 70s and 80s have seen the birth of liver transplantation. In the same way, laparoscopic surgery has been introduced quite late in hepatobiliary units but has nowadays gained acceptance as standard of care for many liver operations, given its superiority in terms of perioperative complications and its similar long-term outcomes.

Yet not all liver resections are the same and some liver segments are particularly difficult to approach in laparoscopy. In particular, the superior liver segments (including SIVa, SVII and SVIII) represent a surgical challenge and their laparoscopic resection is considered a “technically major” hepatectomy (1–3). According to the Louisville conference consensus and Iwate criteria, laparoscopic hepatectomies may be considered major on the basis not only of the amount of resected parenchyma but also of the technical complexity of the operation (1–3). Although more advanced surgical skills are required to perform anatomical resection, given the surgically hostile location of superior lesions, laparoscopic parenchymal-sparing resections are still considered technically major.

Laparoscopic anatomical SVIII resection (LASVIIIR) is considered by many the most difficult of all. LASVIIIRs are demanding due to a number of anatomical characteristics. SVIII’s position high in the abdomen, under the diaphragm, restricts comfortable access with laparoscopic instrumentation resulting in suboptimal control of dissection and view of the surgical field. SVIII is embedded between the right hepatic vein, middle hepatic vein, and inferior vena cava (IVC). This intimate relationship with main hepatic veins (HVs) and the IVC requires exposure of these major vascular structures, with intrinsic risk of life-threatening haemorrhage. Furthermore, the SVIII portal pedicle (G8) is deep-seated within the hepatic parenchyma and no external landmarks exist to guide dissection. Additionally, SVIII anatomy is so complex and variable that its understanding is still incomplete and no current classification can consistently explain individual cases. Latest evidence suggests that the classical right anterior tertiary division into a caudal SV and a cranial SVIII described by Coinaud only accounts for 50% of cases (4–6).

In 25% of cases, the right anterior portal pedicle (RAPP) branches into two independent segments structured in a ventral (medial) and dorsal (lateral) configuration. In these patients, a conventional “SVIII” does not actually exist. In another 15% of cases, the RAPP has a trifurcation, with a caudal SV and two ventro-cranial and dorso-cranial segments. The latter configuration theoretically permits anatomical resection of only the cranial or ventral independent portion of SVIII. This ventro-dorsal sub-segmentation is also seen in an undefined percentage of patients with a “classical SVIII configuration” due to branching of quaternary portal pedicles, and these too are amenable to sub-segmentectomy. In 10% of cases, the actual configuration does not fall in any of these main patterns. New insights are constantly being reported, and these in turn improve our surgical techniques and our patients’ outcomes.

On these premises, it is easy to imagine that at the moment there is no standardised approach to LASVIIIR, and many authors have proposed new and different technical approaches in an effort to overcome the many surgical hardships.

The aim of this review is to describe all the various surgical techniques and tip and tricks for successful LASVIIIR and laparoscopic non-anatomical SVIII resection (LnASVIIIR).

## 2 Methods

A systematic literature search using the PubMed database was performed by three authors (AA, BS, LS) to investigate current surgical techniques for laparoscopic SVIII resection. Keywords used were “segment 8”, “segment VIII” and “liver laparoscopic”. Any relevant article was included, independent of study type, including systematic reviews, randomised and non-randomised trials, case series, case reports, video vignettes, and letters to the editor. References of each selected article were screened to identify additional potentially missed relevant articles. Reports in language other than English were excluded.

When articles featured videos, they were all carefully watched by three independent authors (AA, BS, LS) in an effort to obtain complete understanding of technical details. To better describe the surgical technique, the POSEIDON chart encompassing all main features of laparoscopic hepatic resections was purposefully designed (**Table 1**) and applied to all articles referring to isolated SVIII resection (**Table 2**). Articles were classified by three independent authors (AA, BS, LS). Disagreement was resolved by discussion, and if consensus on a given surgical technique was not reached, a fourth senior author was consulted (GT). Given that most selected articles are case reports, video vignettes or small case series leading to small numbers and high heterogeneity, results are reported as a narrative review.

**Table 1:** The POSEIDON chart.

**PATIENT POSITION (one answer/more than one/more than one)**		
** 1. Supine**	a. Legs closed	α. Surgeon standing between legs
** 2. Semi-left lateral**	b. Legs opened	β. Surgeon standing at right side
** 3. Full left lateral**	c. Right arm in swimming position	γ. Surgeon standing at left side
** 4. Semi-prone**	d. Right flank pillow	δ. Surgeon shifting position
** 5. Prone**		
**PRINGLE MANOEUVRE (one answer)**		
** 1. Intracorporeal**	a. Intermittent during transection	
** 2. Extracorporeal**	b. “à la demand”	
**LAPAROSCOPIC VIEW (more than one)**		
** 1. Ventral**		
** 2. Caudal**	a. No intercostal trocar	
** 3. Cranial**	b. One or more intercostal trocar	
** 4. Lateral (L/R)**		
** 5. Dorsal**		
**GLISSONEAN PEDICLE APPROACH, DEMARCATION, SECTION (one answer/more than one/more than one)**		
** 1. Intrafascial**		α. Clips
** 2. Extrafascial hilar with/without minimal parenchymal disruption**	a. Ischemic demarcation	β. Ligation γ. Suture
** 3. Extrafascial transfissural**	b. ICG negative staining	δ. Stapler
** 4. Extrafascial transparenchimal with major parenchymal disruption**	c. ICG positive staining	εε. Energy Device (**U**ltrasound, **R**adiofrequency, **M**ixed)
**HEPATIC VEIN APPROACH, DISSECTION/EXPOSURE, SECTION (one answer/one answer/more than one)**		
** 1. Extrahepatic isolation and control**	a. Cranio-caudal dissection or root to periphery	α. Clips
** 2. Intrahepatic**	b. Caudo-cranial dissection or periphery to root	β. Ligation
		γ. Suture
		δ. Stapler
		ε. Energy Device (**U**ltrasound, **R**adiofrequency, **Mi**xed)
**DEEP PARENCHYMAL TRANSECTION (more than one)**		
** 1. Monopolar**		
** 2. Bipolar**		
** 3. Ultrasonic dissector**		
** 4. Energy device (Ultrasound, Radiofrequency, Mixed)**		
**CUT SURFACE HAEMOSTASIS (more than one)**		
** 1. Monopolar**		
** 2. Bipolar**		
** 3. Clips**		
** 4. Stiches**		
** 5. Argon beamer**		
** 6. Glue/patches**		
**SPECIMEN EXTRACTION (more than one)**		
** 1. Trocar enlargement**		
** 2. Pfannenstiel**		
** 3. Other service incision**		
** 4. Natural orifices**		

**Table 2:** Techniques for laparoscopic isolated SVIII resection.

First author	Year	Salient Technique features	Poseidon chart	Cases (n°)	Indication(s) (%)	Mean blood loss (mL)	Mean operative time (minutes)	Post-operative complications (Clavien–Dindo)	R0 resection (%)	Long-term oncological outcomes
*Berardi* (7)	2019	Hilar Glissonean-first approach and ICG fluorescence	P1aβ O2a S2a E2abδ I2bαε D234 O//N1	1	HCC	261	420	Grade I: 100% Grade II: 0% Grade III: 0% Grade IV: 0% Grade V: 0%	100%	//
*Boggi* (8)	2015	Robotic	P1bγ Ono!! S/a E//αβγ I//αβγ D12 O//N1	6	//	//	//	Grade I:// Grade II:// Grade III:// Grade IV:// Grade V: 0%	100%	//
*Giuliani* (9)	2015	Caudo-cranial approach	P1bα O2b S/a E//I2bαδ D234 O6 N2	23	HCC: 48% CLRM: 30% Benign lesions: 9% “other”: 13%	//	//	Grade I:// Grade II:// Grade III:// Grade IV: 0% Grade V: 0%	//	//
*Inoue* (10)	2017	Intercostal trocars, caudo-cranial dissection	P3aβ O2a S4b E1b αδ I2/αδ D34 O/N1	29	HCC/ICC: 45% Others: 55%	50	150–183	Grade I:// Grade II:// Grade III:// Grade IV: 0% Grade V: 0%	91%–100%	//
*Ishizawa* (11)	2012	Intercostal trocar, lateral approach, cranio-caudal dissection	P3cδ O1b S4b E1/αε I1aαε D23 O346 N/	4	//	100–1,100	132–240	Grade I:// Grade II:// Grade III:// Grade IV: 0% Grade V: 0%	//	//
*Jang* (12)	2017	Hilar Glissonean-first approach, intercostal trocar, caudo-cranial	2aβ O2a S24b E2aα I2b/D3 O/N/	1	HCC	600	420	Grade I: 0% Grade II: 0% Grade III: 0% Grade IV: 0% Grade V: 0%	100%	//
*Kim* (13)	2019	Trans-parenchymal Glissonean-first approach	P1b/O1a S2a E4aα I2bα D3 O/N/	1	HCC	80	260	Grade I: 0% Grade II: 0% Grade III: 0% Grade IV: 0% Grade V: 0%	100%	//
*Kim* (14)	2019	Transfissural Glissonean-first approach	P1bβ Ob/S2a E3aα I2bα D3 O/N1	1	Adenoma	30	180	Grade I: 0% Grade II: 0% Grade III: 0% Grade IV: 0% Grade V: 0%	100%	//
*Lee* (15)	2014	Intercostal trocar	P1bδ O1b S4b E//I//D3 O36 N1	2	//	//	//	Grade I: 0% Grade II: 0% Grade III: 0% Grade IV: 0% Grade V: 0%	100%	//
*Lopez-Ben* (16)	2021	Lateral approach, cranio-caudal dissection	P1b O2b S3a E4abα I1aα D34 O//N//	1	CRLM	250	265	Grade I: 0% Grade II: 0% Grade III: 0% Grade IV: 0% Grade V: 0%	//	//
*Martinez-Cécilia* (17)	2017	Diamond technique	P1aδ O2a S1a E//I2bαγ D34 O6 N12	13	CRLM: 54% Other metastases: 46%	191	200	Grade I: 15% Grade II: 8% Grade III:// Grade IV: 0% Grade V: 0%	92%	//
*Ogiso* (18)	2020	v5-guided transfissural Glissonean-first approach	P//O//S2a E3bαε I2bα D34 O//N//	1	HCC	170	458	Grade I: 0% Grade II: 0% Grade III: 0% Grade IV: 0% Grade V: 0%	100%	//
*Ogiso* (19)	2021	v5-guided transfissural Glissonean-first approach	P3γ O//S2a E3aαε I2bαδ D34 O2 N//	1	CRLM	30	436	Grade I: 0% Grade II: 0% Grade III: 0% Grade IV: 0% Grade V: 0%	//	//
*Ome* (20)	2019	Transfissural Glissonean-first approach, caudo-cranial dissection with “back-scoring” vs. cranio-caudal approach	P1aβ O2b S3b E3aα I2aα D3 O//N1	26 (caudal: 7 vs. cranial: 19)	HCC: 62% CRLM: 23% ICC: 12% HCC-ICC: 3%	150 (caudal: 200 vs. cranial: 105%)	363 (caudal: 385 vs. cranial: 318)	Grade I: 0% Grade II: 4% (caudal 0% vs. cranial 4%) Grade III: 8% (caudal 8% vs. cranial 0%) Grade IV: 0% Grade V: 0%	92% (caudal: 71% vs. cranial 100%)	//
*Xiao* (21)	2016	Cranio-caudal dissection	P1 O S2a E1aα I1aαγ D34 O2 N12	3	HCC: 100%	300	370	Grade I: 0% Grade II: 0% Grade III: 0% Grade IV: 0% Grade V: 0%	//	//

HCC, hepatocellular carcinoma; ICC, intrahepatic cholangiocarcinoma; CRLM, colorectal liver metastases.

## 3 Feasibility and Outcomes

The feasibility of laparoscopic isolated SVIII resection has been widely demonstrated in the last 2 decades, although most literature refers to “postero-superior segments” including SVII. In general, SVIII resection has been found to be more difficult compared to resection of other segments, with a longer operative time and an increased conversion rate in some series but with comparable short- and long-term outcomes (9, 22, 23). In fact, a study has shown how SVIII resection is introduced late in the learning curve of a minimally invasive hepatobiliary unit, with a significant increase in its performance with time and experience (24). Compared to open surgery, the benefits of laparoscopic resections have been confirmed for SVIII as well, while not affecting oncological results (23, 25, 26).

## 4 Many Techniques for a Challenging Operation

The technical challenges posed by laparoscopic SVIII resections have stimulated many surgeons in producing important efforts to overcome them in the last decade. This has resulted in the rapid development of a wide range of techniques, each of which has its own rationale, usefulness and suggested indication. The current review is based mainly on case reports or short case series, and no strong evidence can be found in the literature to recommend one technique above the other.

Some common principles are agreed upon by all surgeons whatever the technique employed. These include the encirclement of the hepato-duodenal ligament to prepare for Pringle manoeuvre and maintenance of a low central venous pressure (“monitored” through caval fluctuations) during transection. Intraoperative ultrasound (IOUS) is fundamental to confirming the localisation, dimensions, depth and confinement of the lesion and is used prior to start of dissection by most authors and often regularly exploited to confirm the correctness of dissection planes and distance from the lesion (i.e. surgical margins). Other details are different in each report, as they are much related to each surgeon’s personal experience and preference such as the choice of device for dissection and haemostasis.

The detailed technique described in every article focusing on SVIII resection is summarised in **Table 2**.

### 4.1 Abdominal Approach

#### 4.1.1 Non-Anatomical Resection

**Figure 1:**
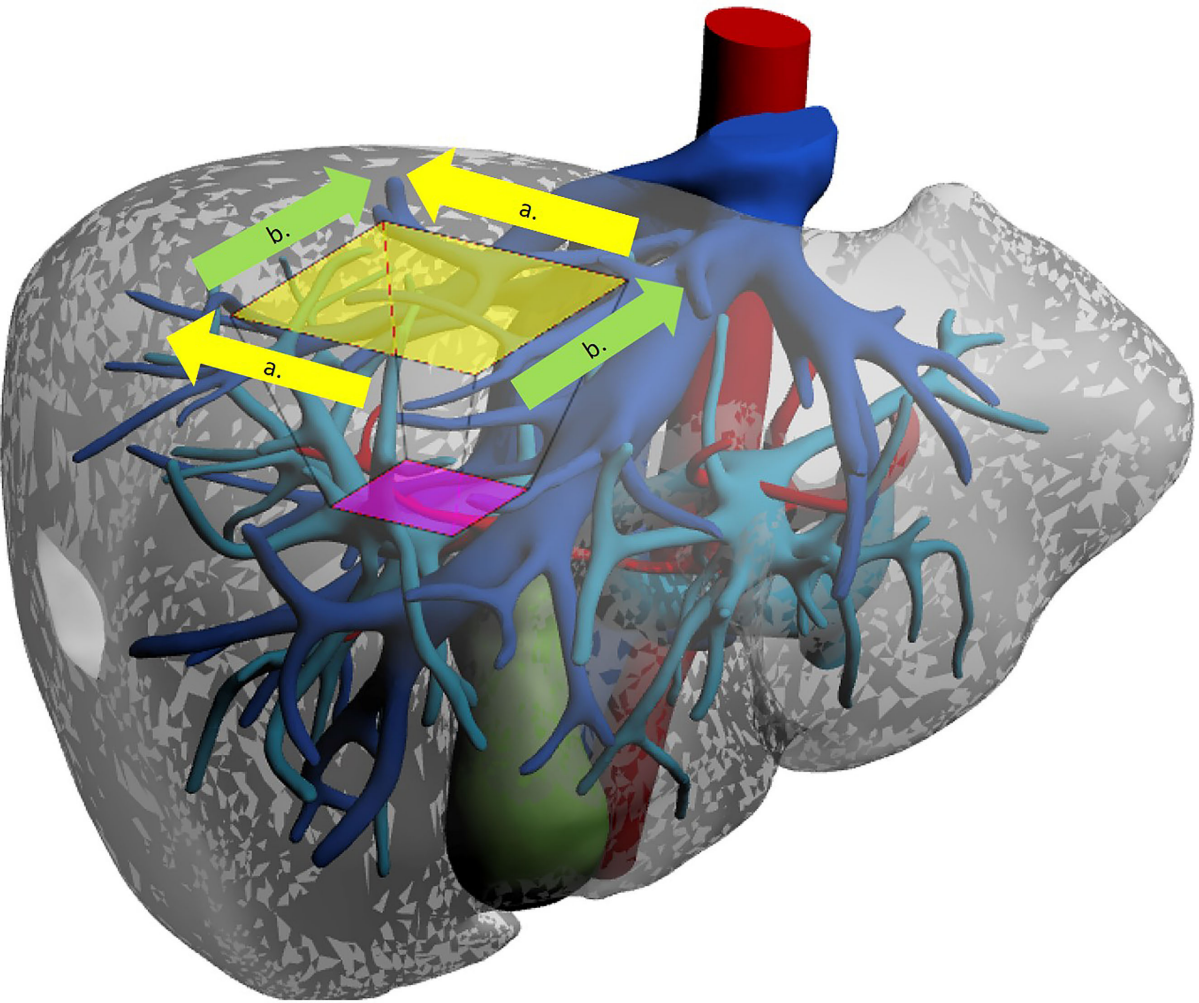
The diamond technique. Operator is placed alternatively at the right or left side. Two perpendicular dissection lines aiming to obtain a semi.diamond shape. a. dissection line from left to tight. b. Dissection line from caudal to cranial.

Given the deep-lying position of the G8 portal pedicle, the non-anatomical resection of SVIII has substantial advantages in terms of technical complexity of the procedure. Nonetheless, the sub-diaphragmatic position confers difficulties even to wedge resections. For this reason, the Southampton group led by Abu Hilal has developed a standardised technique for SVIII wedge resections (17). Their described “Diamond technique” implies that transection is carried out along four linear transection lines which are easier to follow and outline a “diamond” shape (**Figure 1**).

##### 4.1.1.1 Setup

The technique exploits optimal trocar placement to facilitate transection. The patient is placed supine with the surgeon alternating between the patient’s right and left. Four or five cannulas are placed in a reverse-L configuration along the left and lower borders of the tumour.

##### 4.1.1.2 Procedure

Trocar configuration provides accesses that will permit use of the transection device perfectly in line with the ideal transection plane, along every side of the quadrilateral resection. Alignment of the instrument and transection plane ensures a lower risk of convergence towards the specimen especially with deeper dissection. Traction sutures may be placed to help retract margins. An interesting tip to help retraction of the to-be-resected liver comes from a Japanese group (27). They sutured an elastic silicone band to both the liver and the diaphragm, producing tensile strength that automatically uplifted the liver and opened the transection plane. This trick may be helpful as one of the main struggles during LLR is that of obtaining correct counter-traction.

##### 4.1.1.3 Results

Reported results for 13 patients with isolated SVIII lesions were optimal, with 0% perioperative major morbidity and satisfactory oncologic results (92% R0 resection). Long-term outcomes for isolated LnASVIIIR are not available, yet a median recurrence-free survival of 59 months and overall survival of 61 months are reported for LnASVIIIR combined with other segments’ resection (17).

#### 4.1.2 Anatomical resection

Other authors have posed their attention on isolated SVII resections and have gone further in describing techniques for proper anatomical resection. Anatomical resection may bear specific advantages over non-anatomical resection from an oncologic point of view.

First, anatomical resection involves a larger area of liver parenchyma, increasing the chance of obtaining adequate resection margins. Furthermore, removal of the entire involved portal territory has been reported to increase recurrence-free survival, especially in hepatocellular carcinoma with portal invasion and in patients without full-blown cirrhosis (28–31). Finally, remnant liver ischemia has also been shown to be correlated with an increased recurrence rate (32).

Although this has not been clearly demonstrated, better comprehension of anatomy and refinement of the surgical technique should bring to more precise anatomical resections and reduced remnant liver ischemia (32). On the other hand, most HCCs appear on a background of liver cirrhosis, recur anyway and may require repeat resection or be referred for liver transplantation: patients may benefit from parenchymal sparing techniques. In this scenario, sub-segmentectomy (when feasible) may provide an optimal solution.

##### 4.1.2.1 Glissonean-First Approach

There is no superficial anatomical landmark for SVIII; hence, the intuitive approach for anatomical resection is “Glissonean-first”: surgery evolves around the identification and isolation of the SVIII portal pedicle (G8) and its clamping (confirming SVIII ischemia) and division, followed by transection along demarcation margins, along RHV and MHV planes, usually in a caudo-cranial fashion. Venous branches draining in the RHV and MHV are carefully controlled and divided as they are encountered. A major difficulty in performing anatomical SVIII resection is that G8 is buried deep within the parenchyma and there are no landmarks to facilitate its identification. Access to the SVIII pedicle is technically demanding, and a number of ways to obtain it have been developed.

###### 4.1.2.1.1 Setup

The patient is usually placed supine, and the surgeon stands either between legs or on the patient’s left side. A total-abdominal approach is mostly used, but some authors have proposed the introduction of intercostal trocars to improve the angle of surgical instruments relative to the hepatic veins, especially for dissection high in the dome area. The Osaka group, led by Inoue, published their series of 29 patients treated with (n11) and without (n18) intercostal trocars. They report no difference in patient outcomes (including overall complications, R0 resection, hospital stay, blood loss) with the exception of conversion to open surgery: 0% of patients in the intercostal group vs. 38.9% of patients in the “conventional” group (*p* = 0.026). Chiow et al. instead compared patients treated with and without intercostal trocars and found no difference but for hospital stay: median 2 vs. 6 days, respectively (*p* 0.032) (33). Both studies compare their current technique using intercostal trocars with their previous experience, and this represents a serious limitation to interpretation of results as the latter may have been affected by improvements in technical expertise or technology over time (10, 33). Analysing specific intercostal trocar-related complications, Hayashi et al. report their experience with 32 patients with only 1 clinically silent minor pneumothorax (34). A chest drain was left in place after the procedure in only 2 cases due to air infiltration in the thoracic cavity. IOUS application to the Glissonean-first approach is fundamental to reaching G8 in the transparenchymal and transfissural approaches but can also be useful for the hilar approach to guide dissection along Glissonean pedicles.

###### 4.1.2.1.2 Procedure

**Figure 2:**
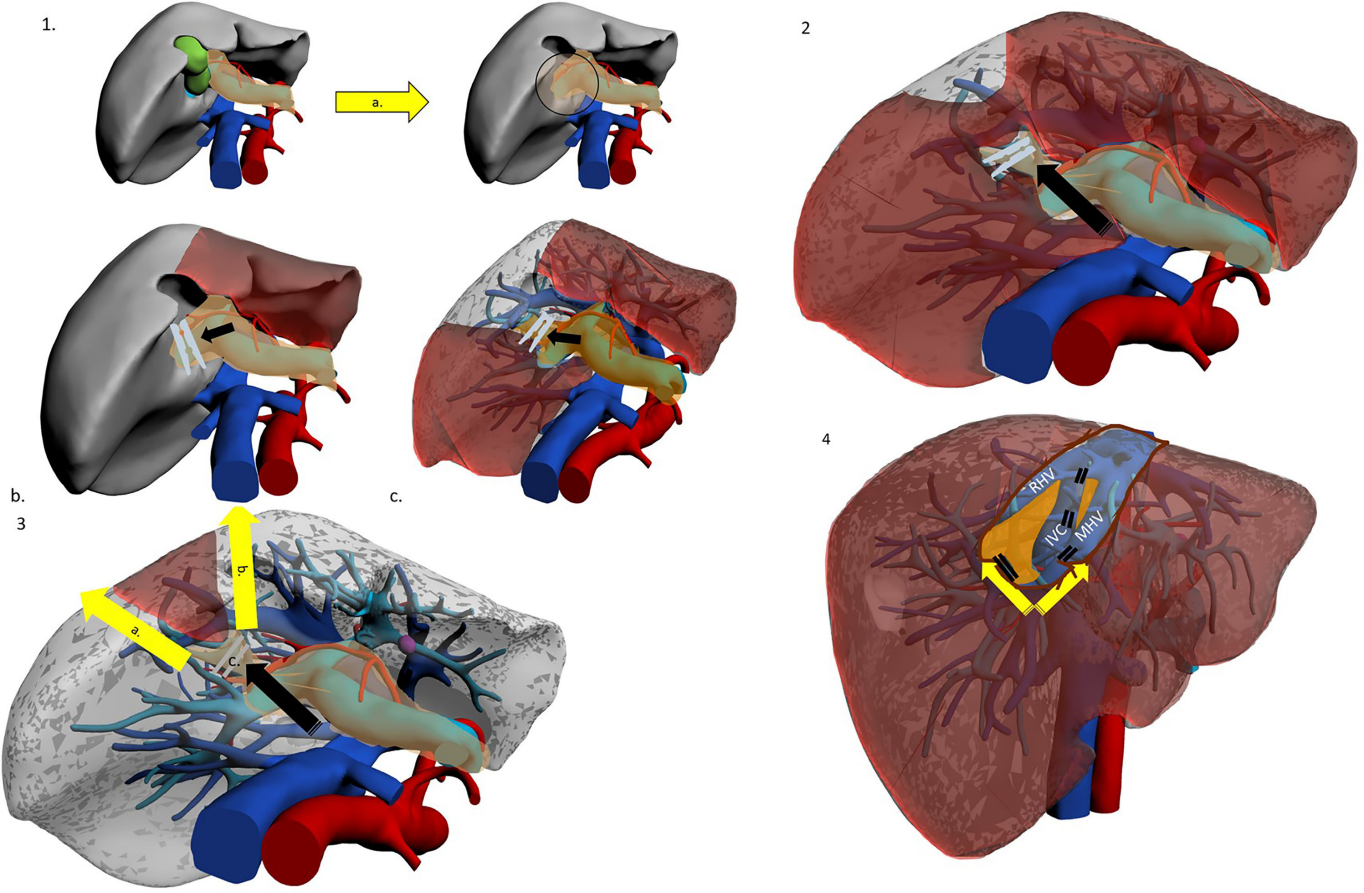
The hilar Glissonean-first approach. Patient in lithotomy or left semi-decubitus position. Possible use of intercostal trocar. Operator is placed at the right side. 1a. Cholecystectomy for better access to right pedicle. 1b. Hilar Glissonean isolation, clamping and demarcation of the right pedicle. 1c. Hilar Glissonean isolation, clamping and demarcation of the right anterior pedicle. 2. Hilar Glissonean isolation, clamping and demarcation of G8 pedicle. 3a.-b. Dissection lines. 3c. Clipping of G8 pedicle. 4a Dissection proceeds in caudo-cranial and medio-lateral direction exposing MHV, IVC and RHV and clipping all their tributaries. 4b. At the end of the SVIII resection MHV, IVC, and RHV are fully exposed.

The first route is hilar: dissection starts at the hepatic hilum with identification of the portal bifurcation (7) (**Figure 2**). The right Glissonean pedicle is followed until its own bifurcation in the right anterior and posterior pedicles. The right anterior pedicle is clamped to confirm its area of perfusion. It is then followed further until its separation into the SV and SVIII pedicles. The SVIII pedicle is identified, confirmed and divided. The approach was described in detail by Jang et al. in 2017 (12). This technique holds the advantage of unequivocal identification of G8 through a structured step-by-step, secure path. When the right and right anterior pedicles are relatively short, this technique may be ideal. Yet navigation of main Glissonean pedicles may not be risk-free and major vascular accidents are always possible. Small branches may also suffer injury, and this in turn may lead to biliary complications such as leakage or delayed biliary stricture. Furthermore, if the intrahepatic course of these two branches is long, the hilar approach may prove difficult and time-consuming.

**Figure 3:**
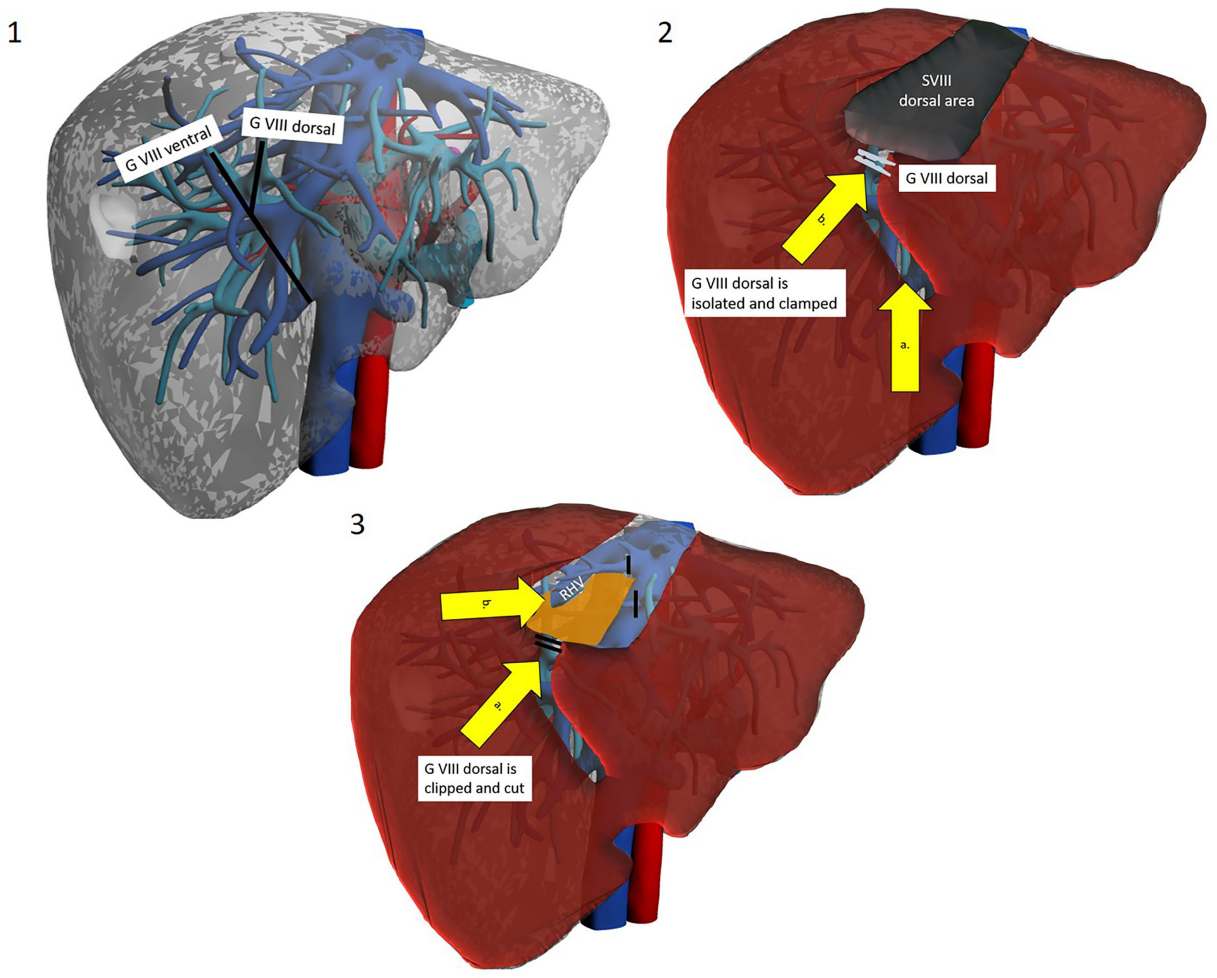
The trasparenchymal Glissonean-first approach. Patient is in lithotomy position. Operator is placed between legs. 1a. After intraoperative ultrasound the projection’s lines of right anterior Glissonean pedicle, G VIII ventral and G VIII dorsal are marked on the liver surface. 2a. The liver surface along the right anterior pedicle is opened with an energy device and CUSA. 2b. Glissonean VIII dorsal pedicle is identified, isolated and clamped to obtain parenchymal demarcation. 3a. Glissonean VIII dorsal pedicle is clipped and cut. 3b. Dissection proceed along the inferior border to reach the posterior boundary of segment VIII that is the RHV.

Kim et al. have proposed a different approach, named “transparenchymal” (**Figure 3**). In this case, the right anterior Glissonean pedicle is identified through IOUS on the ventral liver surface and a small bridge of parenchyma is divided to access the vascular space (13). G8 is then identified, and the operation proceeds with the steps already described. This approach has some theoretical advantages over the “hilar approach”: it is faster, it involves less parenchymal disruption and most importantly it avoids hilar dissection. The latter can be of particular importance for patients who are likely to undergo further liver surgery (e.g. HCC patients who might suffer recurrence or eventually undergo transplantation) as preserving an intact hilum reduces chances of hilar adhesions and reduces difficulty of dissection and risks of vascular/biliary accident. This approach may theoretically be simplified by exploiting Sugioka’s Laennec’s capsule-based technique to gain easy access to the right anterior Glissonean pedicle with no or minimal parenchymal disruption, yet it has never been described except by Sugioka himself in an untranslated Japanese article (35, 36). A recent video article describes in detail this approach to the Glissonean pedicle (37).

**Figure 4:**
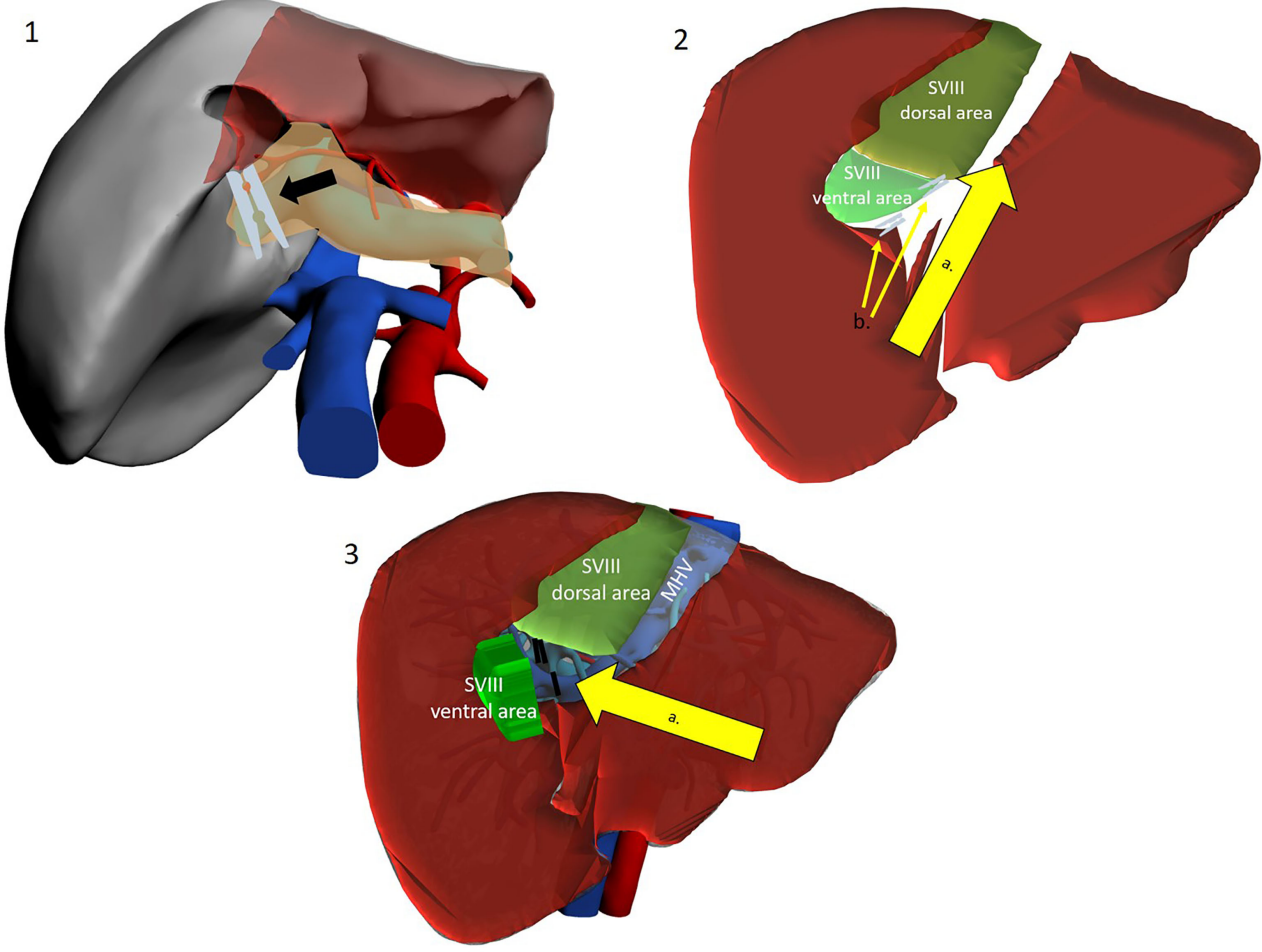
The transfissural Glissonean-first approach. Patient is in lithotomy position. Operator is between legs. 1. Hilar Glissonean isolation and clamping of the right pedicle with demarcation along Cantlie’s line. 2a. Opening of the main portal fissure. 2b. Isolation, clamping and demarcation of the SVIII ventral and dorsal pedicle. 3a. Clipping and section of the SVIII ventral pedicle. Resection along the demarcation line.

A third possibility is represented by the transfissural approach (38) (**Figure 4**). The main hepatic fissure is marked on the liver surface, using the MHV as the main IOUS landmark or alternatively by clamping the right Glissonean pedicle (14). Parenchyma is divided at this level, ultimately opening the liver like a book, eventually landing on the MHV. This procedure helps in the identification of G8 (which may still be troublesome due to anatomical variations) and most of all helps with the ensuing dissection along the MHV and anterior border of the IVC, guaranteeing a comfortable access. Ogiso et al. have gone a little bit further, with their valuable anatomical and surgical studies. Their work, with 3D reconstruction and simulation modelling on CT images of 40 patients, suggests that G8 can be found quite constantly behind an SV branch of the MHV (v5) (39). Therefore, they have standardised a “reliable and reproducible” Glissonean transfissural v5-guided technique for anatomical SVIII resections (18). Their main point of reference is v5, identified with IOUS before start of dissection and constantly reassessed during dissection of the main fissure. Once v5 is reached and sacrificed, easy access to G8 will be acquired. The authors state that this renewed anatomical knowledge may indeed be key to dissemination of laparoscopic SVIII resection. The same group has found this technique to be suitable even when dealing with large tumours at the hepatocaval confluence (19). They report that complete opening of the main fissure facilitated dissection of the dorsal side of the mass.

The Glissonean approach ensures radical, anatomical resection of SVIII along correct planes by meticulous identification of vascular inflow. A further advantage is that sub-segmental resection can be achieved: dissection of vascular structures can be extended to permit identification of sub-segments/cone units. A preoperative accurate study of CT images and possibly 3D reconstruction is clue to planning these operations. When a lesion is located within anatomical borders of a sub-segment, its resection may be ideal in its dual scope of removing the whole tumour-burdened portal territory and widening margins while conserving parenchyma. Both dorsal and ventral SVIII resections have been reported by Kim (13, 14) (**Figures 3**, **4**).

###### 4.1.2.1.3 Results

**Table 2** summarises results for isolated LASVIIIR. Data are available for 71 patients for isolated LASVIIIR with a Glissonean-first approach (7–10, 12–14, 18, 19). Results are encouraging with major morbidity reported only in 3% (2 patients with bile leak), no mortality. Margin status was available in 47 patients and R0 resection achieved in 91%. No long-term oncologic results are available, for any indication.

Whatever the technique to access G8, the Glissonean approach carries some drawbacks: possible injury to small branches and consequent biliary complications, division of SV venous drainage and consequent SV congestion and the difficult dissection of RHV, MHV and anterior IVC surface from below—the caudal laparoscopic view.

##### 4.1.2.2 Cranio-Caudal Approach

**Figure 5:**
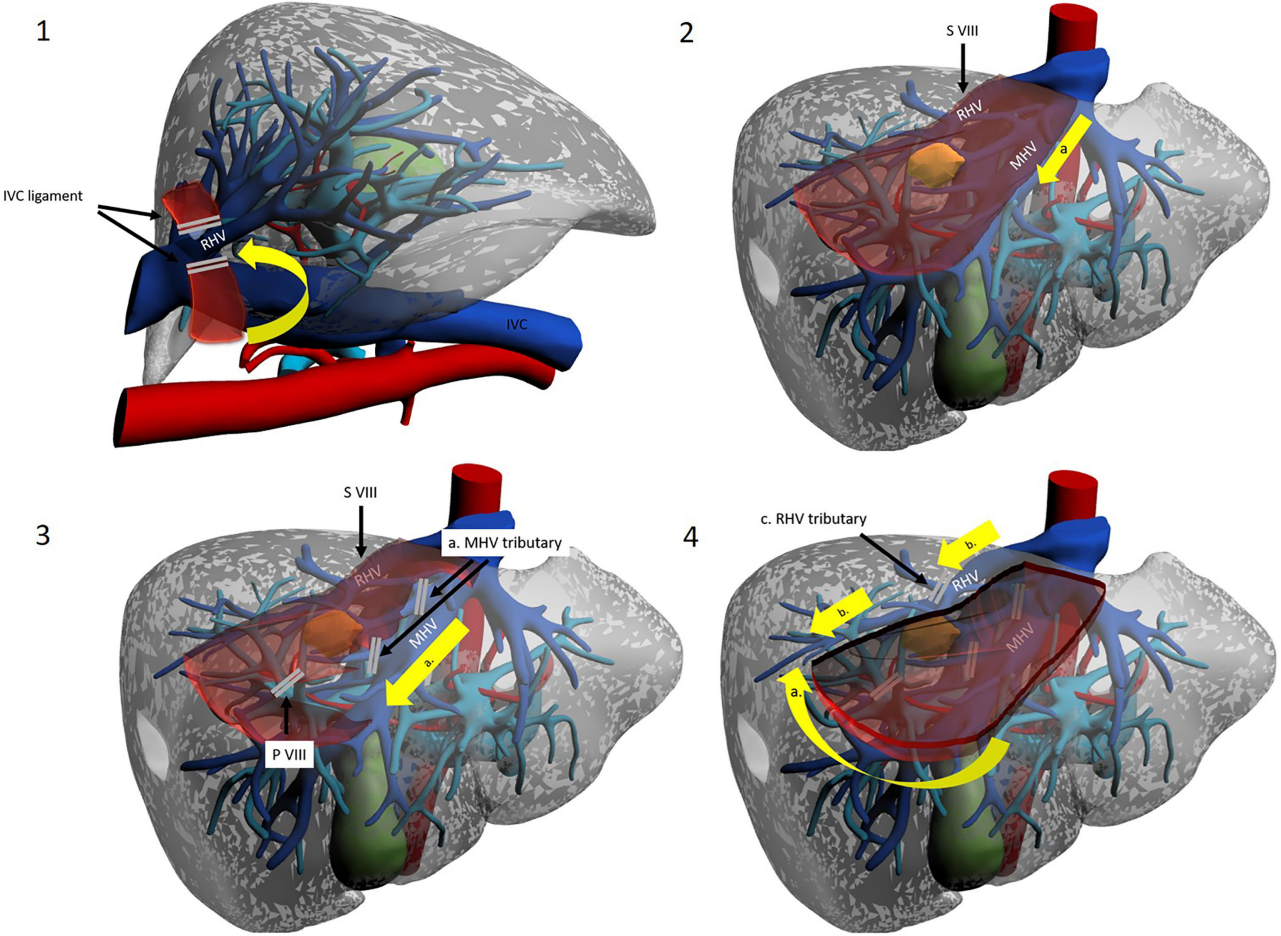
The cranio-caudal approach. Operator is placed on the right side. 1a. Mobilization of the right liver. 1b. Division of the IVC ligament. 1c. Isolation and taping of RHV. 2a. Dissection line along MHV from head side. 3a. Control of the MHV tributaries draining segment VIII. 3b. Division of the S VIII portal pedicles. 4a. Dissection of the inferior border along the demarcation line. 4b. Dissection line along RHV from head side to foot side. 4c. Control of the RHV tributaries draining segment VIII.

**Figure 6:**
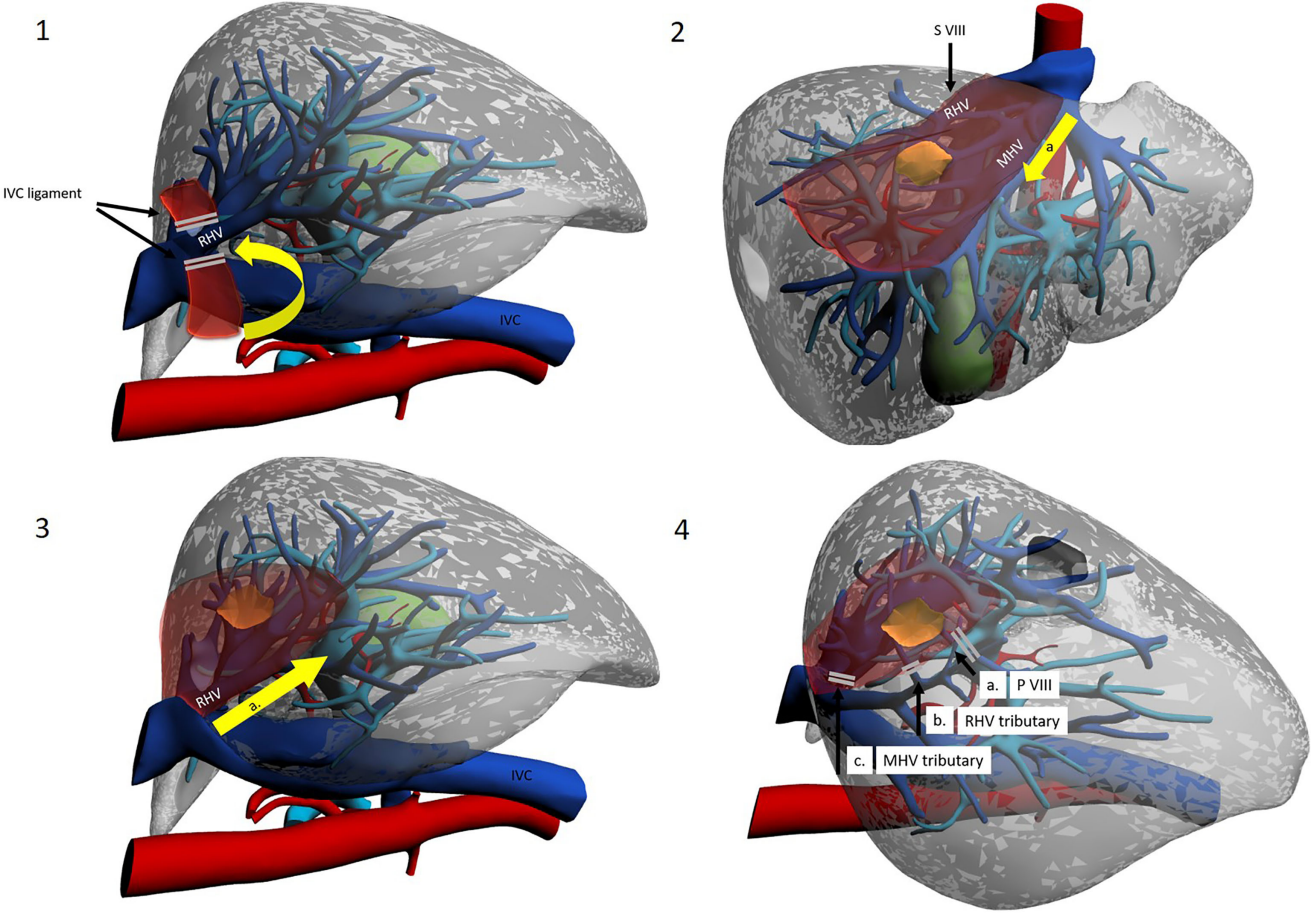
Use of intercostal trocars for cranio-caudal dissection with the lateral approach. Left lateral decubitus. Operator is placed on the right side or between legs. 1a. Mobilization of the right liver. 1b Division of the IVC ligament. 1c Isolation and taping of RHV. 2a. Dissection line along MHV from root to periphery. 3a. Dissection line along RHV from root to periphery. 4a. Division of the S VIII portal pedicles. 4b. Control of the RHV tributaries draining segment VIII. 4c. Control of the MHV tributaries draining segment VIII.

A different, cranio-caudal approach was proposed to make up for the shortcomings of caudo-cranial dissection. This approach is interchangeably referred to in the literature as “cranio-caudal”, “head-side” or, by some authors, “ventral” (40) (**Figures 5**, **6**).

###### 4.1.2.2.1 Setup

The patient can be placed either supine or in the left lateral decubitus position to facilitate access to the right hemi-liver. Right subcostal trocars are placed, with the surgeon standing alternatively between legs and on the right side of the patient. Although proponents report an easier dissection, the cranio-caudal approach still results technically difficult when performed with instruments that are coming from below, approaching veins in a parallel direction. This may increase the chances of injury to branching points of the vein. To circumvent this obstacle, a further technical modification has been introduced. Ishizawa et al. were the first to describe the use of intercostal trocars to obtain a lateral view which may be more convenient to approach the hepatic veins in a cranio-caudal fashion (11) (**Figure 6**). These trocars also allow for a non-parallel approach to major venous dissection. The patient is placed in the left lateral decubitus position, and insertion of intercostal trocars is performed under direct vision. Three main techniques are described: in any case, much attention should be given to place the trocar centrally in the intercostal space to avoid intercostal vessels, which run along the inferior costal margin. The first is to use trocars through the ribs but below the diaphragm, with direct access to the abdominal cavity. These trocars though will be quite low and be less helpful in the dissection. Another technique used for example by Ishizawa et al. is to compress the diaphragm against the chest wall with a forceps to exclude the lung from the pleural space around the trocar insertion site, without deflating the lung (11). The third option is to use an optical trocar to gain access to the thoracic cavity and then pierce through the diaphragm, while collapsing the right lung (10). Insertion is preferentially preceded by IOUS to visualize the target lesion and by liver mobilisation on demand (34). Two or three intercostal trocars may be placed, either in the same or in different intercostal spaces (usually between the 6th and 10th). Ports are usually 5 mm, to minimize complications, as the intercostal space may be as small as 15 mm and placing a 10–12-mm trocar may increase bleeding risk (10). Most authors advocate the use of balloon ports so that the inflated balloon may pull the diaphragm against the chest wall, enlarging the field of view. After hepatectomy, aspiration of gas in the pleural space is important, and so is suturing of the phrenic incisions from below. Ome et al. conclude that the use of intercostal trocars makes the cranial approach safer and more feasible (20). It seems reasonable to consider the practice safe, yet a relevant limitation of this procedure is that the hepatobiliary surgeon may not be familiar with intercostal trocar placement, increasing the likelihood of thoracic complications. Furthermore, the “needless” violation of a different anatomical district gives concerns for oncological catastrophe. IOUS is vital for the cranio-caudal approach and represents the surgeon’s companion along the whole operation. In particular, as most SVIII dissection is carried out before G8 division, it cannot be based on ischemic demarcation but is based mostly on the direction outlined by IOUS detection of RHV and MHV.

###### 4.1.2.2.2 Procedure

Surgery starts with right liver mobilisation, and the RHV is fully exposed from all sides by division of the right hepatocaval ligament. Dissection and encirclement of RHV and MHV roots usually follow. Extrahepatic control is not strictly necessary, and some authors do not perform this manoeuvre, although the potential vascular risk has been an object of debate (41). Once vascular outflow control is achieved, and after IOUS-guided marking of the venous plane, transection follows the hepatic veins down into the parenchyma, exposing the bare venous surface. Dissection may either start from the MHV, effectively opening the main hepatic fissure until the root(s) of G8/G8 branches (11, 15, 21), or begin from the RHV and then proceed medially along the anterior IVC surface (16). The latter approach is sometimes referred to as the “lateral” or “dorsal” approach (**Figures 5**, **6**). G8 is identified late in the operation when at least one side of the resection has been conducted. Nonetheless, Ome et al. describe their MHV-first approach as “Glissonean” as they divide G8 before initiating other transection planes (20). Supporters of this technique argue that beginning dissection from above provides an easier plane to follow as it is “clear and shallow” and safer as hepatic veins have thick walls and fewer branches at this latitude and exposure of the IVC are also facilitated (21). Furthermore, when dissecting HVs from the root, finding a plane to preserve Laennec’s capsule seems possible, thereby conserving HV parietal resilience (40). Furthermore, due to the anatomical relationship between main hepatic veins and their branches (conflating at acute angles inferiorly), a caudal approach is prone to procuring “split-injuries” while a cranial approach is more likely to produce “pulled-up” injuries, which are considered easier to control, with simple pressure (40). Honda et al. and Ome et al., who use a caudal approach in their routine practice, suggest that using the instrument tip in a back-scoring (root to periphery) fashion may be a good trick to reduce/avoid split injuries (20, 42). Some authors argue that the transection line is easily kept blood-free as blood flows inferiorly by gravity. If haemorrhage from a hepatic vein does occur, decreasing airway pressure (by temporarily pausing ventilation) helps in reducing central venous pressure, and this aids bleeding control (43). Finally, outflow control coupled with the standard Pringle manoeuvre allows for greater confidence in haemostatic control. On the other hand, late G8 control and dissection along landmark yet unconfirmed planes may lead to less precise anatomical resections and to increased remnant liver ischemia. This may be particularly true when dealing with the 25% of cases where the classical right anterior separation into a cranial SVIII and a caudal SV is replaced by a ventro-dorsal tertiary branching (4, 5). Lopez-Ben et al. have also reported the possibility of SVIII dorsal resection with a lateral approach as G8 dorsal can be found in proximity of the RHV, although there is no clear landmark and G8dorsal identification may be problematic (16).

###### 4.1.2.2.3 Results

**Table 2** summarises results for isolated LASVIIIR. Data are available for 27 patients for isolated LASVIIIR with a cranio-caudal approach (11, 16, 20, 21). Results appear superlative with no morbidity nor mortality. Margin status was available only in 19 patients and R0 resection achieved in 100%. No long-term oncologic results are available, for any indication. Ome et al. published a very interesting study comparing 7 and 19 patients treated with the caudo-cranial and cranio-caudal approaches, respectively (20). There were no significant differences between groups, although numerosity is low. Interestingly, two patients in the caudal approach had R1 resection and two had postoperative bile leak while all patients in the cranial group had R0 resections and no biliary complications. The authors report their impression of a cranial approach facilitating obtainment of free surgical margins and reducing biliary injury (either direct or ischemic).

Theoretically, the cranio-caudal hepatic vein dissection is not necessarily an alternative to a Glissonean-first approach. The two approaches could be combined with G8 controlled followed by root-to periphery dissection, although this has not yet been described for laparoscopic SVIII resection to the best of our knowledge.

### 4.2 Transthoracic Approach

Finally, some authors have described the possibility of trans-thoracic resection of SVIII lesions. The rationale for this is mainly that dome lesions, being directly sub-phrenic, may be more easily approached from above, facilitating minimally invasive resection of lesions that would otherwise deserve open surgery. This may be particularly true when trans-abdominal access is rendered troublesome by adhesions due to previous surgical procedures, especially when multiple previous hepatic resections have been performed. Transthoracic resections are wedge-type almost by definition, and no anatomical resection has ever been described. Furthermore, thoracoscopic experience is needed for such operations, which is not common among hepato-biliary surgeons and therefore combined intervention with thoracic surgeons may be required.

#### 4.2.1 Setup

The patient is placed in left lateral decubitus. The adopted technique involves intercostal thoracoscopic access and localisation of the dome lesion by means of a laparoscopic IOUS probe placed on the superior diaphragmatic surface.

#### 4.2.2 Procedure

The procedure proceeds with incision of the diaphragm above the lesion and its resection from a strategic point of view. The diaphragm must then be suture-closed/reconstructed. The greatest limitation is lack of adequate vascular control. Different methods have been employed to make up for this perilous drawback. Qin et al. used transthoracic radiofrequency ablation in an effort to minimize bleeding risk from the transection plane which then run on ablative margins (44). This may also help obtain tumour-free margins. Zhu et al. propose instead a combined approach, with a minimal laparoscopic phase in which a Pringle manoeuvre is obtained followed by a safer trans-thoracic resection (45).

#### 4.2.3 Results

Only a few case reports are present in the literature (44–48). Data are available for 5 cases. Mortality was nil, and morbidity was 20% (1 pleural effusion, resolved with drainage). R0 resection was obtained in 100% of cases but was available only in 2. Oncological outcomes are available for 2 patients who remained disease-free 8 and 24 months after HCC resection (45).

*Advantages and disadvantages of the main techniques reviewed are summarised in* **Table 3**.

**Table 3:** Theoretical advantages and disadvantages of main approaches.

Approach	Advantages	Disadvantages
***Diamond technique***	• Lower risk of convergence towards the specimen especially with deeper dissection → increases R0 resections	• Non-anatomical
***Glissonean-first—hilar***	• Unequivocal identification of G8 through a structured step by step, secure path	• Biliary complications • Time consuming • Hilar adhesions in eventual second resection • Difficult dissection of RHV, MHV and anterior IVC surface from below
***Glissonean-first—transparenchymal (minimal parenchymal disruption)***	• Fast • Little parenchymal disruption • Avoids hilar dissection	• Difficult dissection of RHV, MHV and anterior IVC surface from below
***Glissonean-first—transfissural***	• Comfortable access to MHV and IVC • Facilitates dissection of large masses • V-5 approach reproducible	• Division of SV venous drainage and consequent SV congestion • Large parenchymal disruption • Difficult dissection of RHV, MHV and anterior IVC surface from below
***Cranio-caudal***	• Easier plane to follow • Decreased chance of split injuries • Outflow control • Increased R0 resections • Reduced biliary injury	• Late G8 control • Dissection along unconfirmed planes→ less precise anatomical resections → increased remnant liver ischemia
***Intercostal trocar use***	• Lateral view → non-parallel approach to major venous dissection • Reduced conversion rates	• Thoracic complications→ pneumothorax, bleeding • Unfamiliar to the abdominal surgeon • Needles violation of different body cavity → oncological issue?
***Transthoracic***	• Avoids adhesions in the abdomen → increases minimally invasive approach success in patients with abdominal adhesions	• Non-anatomical • Difficult bleeding control if Pringle is not performed • Unfamiliar to the abdominal surgeon

## 5 Technological Innovations: Utility and Implementation

Robotic surgery has so far found limited applications in LASVIIIR, yet it may represent an important innovation to overcome some of the technical “struggles” dealt with in laparoscopy. The endo-wrist features of the robotic platform may prove important as LASVIIIR demands following curvilinear resection planes, one of the major technical difficulties in traditional laparoscopy. On the other hand, the commonly used current robotic platforms do not feature a specific liver-parenchyma transection device, so that only the crush-clamp technique is available. Laparoscopic dissection devices can be used by the assistant surgeon at the table, but this diminishes advantages of the robotic approach. As remarked by Boggi et al., only when leading companies will invest in liver surgery will we see the real potential of this technology (8). So far, the feasibility of robotic isolated LASVIIIR has been demonstrated by Boggi et al. who reported 6 cases, without complications and 100% tumour-free resection margins (8). In particular, they used a caudo-cranial Glissonean-first approach. Larger series would be needed to confirm safety and results of robotic LASVIIIR.

Use of indocyanine green (ICG) may prove useful for LASVIIIR. In particular, it may better distinguish ischemic liver and therefore be particularly useful for Glissonean-first approaches. In fact, as the liver uptakes ICG very quickly, functioning parenchyma will fluoresce while the ischemic area will be well demarcated. This will probably be most advantageous in the deeper parts of dissection where ischemic demarcation is not as easy to detect as on the liver surface. Given that inter-segment boundaries follow curvilinear planes, this may prove particularly useful in achieving real anatomic resection and reduce remnant liver ischemia (49).

A further application of ICG that could be an adjunct to all approaches is its administration 2 weeks prior to surgery. Japanese groups have demonstrated that fluorescence, given that it is preferentially retained in tumour tissue, will reveal tumour position with clarity (reported 100% sensitivity and high specificity) (7, 50, 51).

## 6 East vs. West: Insights From Different Perspectives

Most studies on isolated SVIII resection come from East Asia. Contributions from the Western world include the Diamond technique and robotic-assisted experiences (8, 17). Most LASVIIIR conducted in the West adopted a caudo-cranial approach while only Lopez-Ben et al. reported a case of cranio-caudal dissection for SVIII dorsal resection and reported the close contact between G8 dorsal and RHV (16). The Eastern world has been more inventive, with ideation and wider implementation of the cranio-caudal approach, the introduction of intercostal trocars and more detailed descriptions of the hilar, transparenchymal and transfissural approaches to G8, including the v5-guided technique (18).

## 7 Authors’ Perspective: Proposal of a Treatment/Approach Algorithm

**Figure 7:**
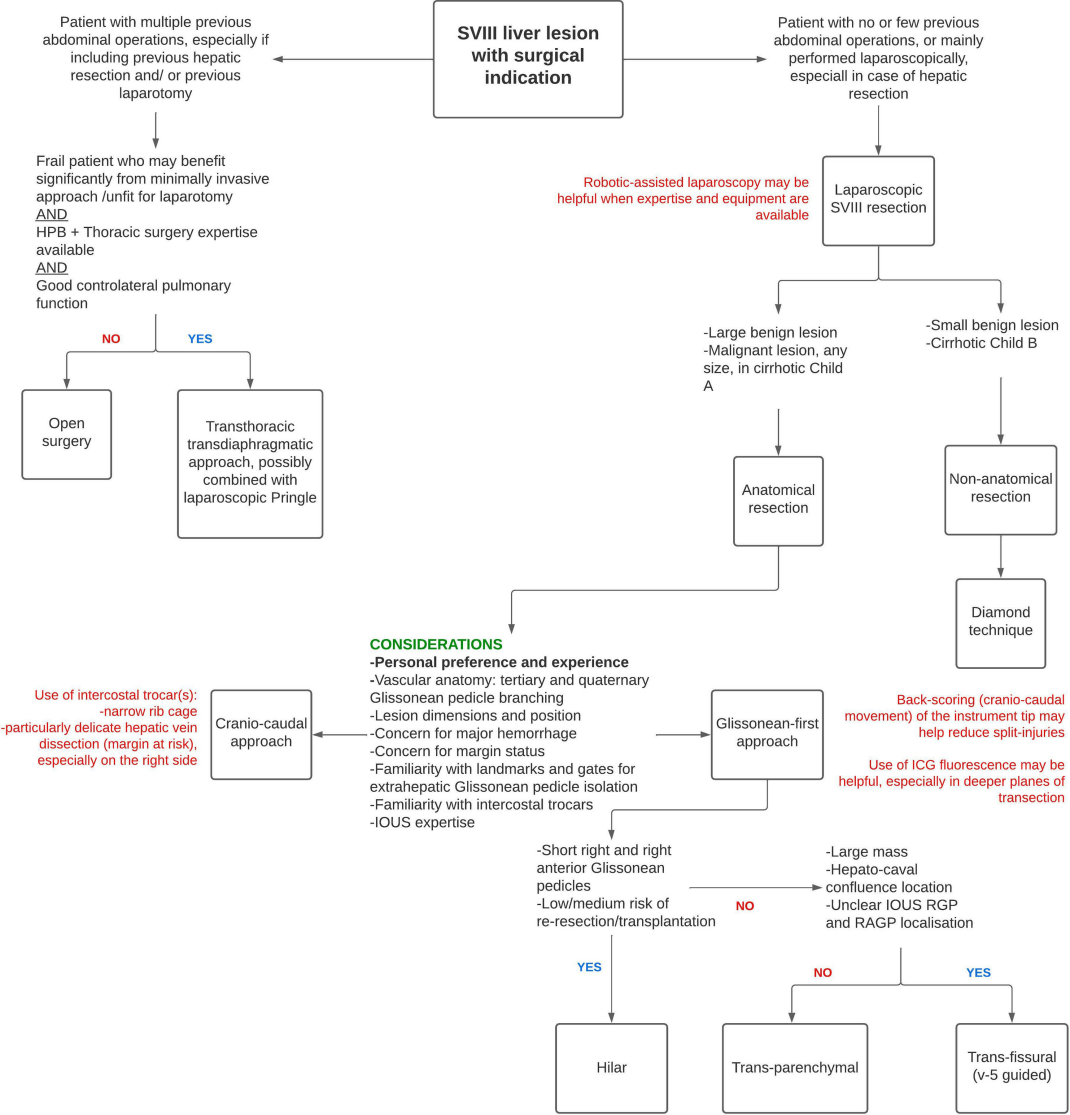
Proposal of a treatment algorithm for tailored-approach to isolated SVIII resection.

Based on the literature discussed above and their centre’s experience, the authors have developed a treatment algorithm to guide the tailoring of the surgical technique to the specific patient–lesion–expertise combination (**Figure 7**). However, given the low level of evidence, this scheme should be considered only in highly experienced, tertiary centres and patients informed that long-term oncological outcomes for isolated LASVIIIR are currently unavailable.

Patients who have undergone previous multiple abdominal surgeries, especially including previous hepatic resection or laparotomy, should probably not be considered for laparoscopic SVIII resection. These patients should be scheduled for open surgery or, especially when frail and only if specific expertise is locally available, transthoracic resection may be taken into consideration. Patients scheduled for a minimally invasive approach can undergo LnASVIIIR for small benign lesions or when liver conservation is a necessity, such as in Child–Pugh B cirrhotic patients. When considering LASVIIIR, a cranio-caudal approach should probably be favoured when obtaining an R0 resection is paramount, if expertise with this technique is available. Use of inter-costal trocars is suggested, especially in patients with a deep hypochondrium and/or a delicate HV dissection is predicted (e.g. margin at risk) especially on the RHV, where the disadvantage due to the instrument-HV angle provided by trans-abdominal trocars is most significant. A Glissonean-first approach can provide a more anatomical resection and possibly less liver remnant ischemia, an important consideration in cirrhotic patients. When this approach is chosen, back-scoring is suggested and ICG use may also be helpful, particularly in deeper transection planes. When the right and right anterior Glissonean pedicles are short and the patient is at low risk for re-resection or transplantation, a hilar approach is preferable. Otherwise, a transfissural v5-guided approach can be undertaken in patients with large masses, hepatocaval confluence localisation or unclear visualisation of right Glissonean pedicles, while a transparenchymal approach should be sought in all other cases.

## 8 Conclusions

Laparoscopic SVIII resection remains a challenging operation, yet its feasibility has been widely demonstrated. Long-term oncological outcomes are still lacking. Many surgical techniques have been developed each with its own benefits and pitfalls: no evidence exists to recommend one over another. The diamond technique may facilitate wedge resections. The Glissonean-first approach may result in the most anatomically precise resections and is key to performing sub-segmentectomies. Cranio-caudal hepatic vein dissection may help in avoiding severe intraoperative bleeding. Intercostal trocars and the lateral view may enhance feasibility of the cranial approach and increase safety. A transthoracic approach may be justified in very selected patients, especially those already submitted to extensive prior liver surgery. Overall, the modern laparoscopic hepatobiliary surgeon should master one of these techniques and be familiar with multiple approaches, the ultimate choice of which should be tailored to the individual case to maximise the oncological benefit-safety profile.

## Author Contributions

AA was involved in the study conception, design, and drafting. BS was involved in the study design and drafting. All authors contributed to critical revision of the article for important intellectual content and approved the final version to be submitted.

## Conflict of Interest

The authors declare that the research was conducted in the absence of any commercial or financial relationships that could be construed as a potential conflict of interest.

## Publisher’s Note

All claims expressed in this article are solely those of the authors and do not necessarily represent those of their affiliated organizations, or those of the publisher, the editors and the reviewers. Any product that may be evaluated in this article, or claim that may be made by its manufacturer, is not guaranteed or endorsed by the publisher.
